# Interventional pulmonary procedures and their outcomes in patients with STAT3 hyper IgE syndrome

**DOI:** 10.1186/s12893-023-02193-2

**Published:** 2023-09-23

**Authors:** Seyed Alireza Mahdaviani, Soodeh Ghadimi, Mazdak Fallahi, Seyedeh Atefeh Hashemi-Moghaddam, Zahra Chavoshzadeh, Anne Puel, Nima Rezaei, Mahsa Rekabi, Zahra Daneshmandi, Kambiz Sheikhy, Abolghasem Daneshvar Kakhki, Seyed Reza Saghebi, Saviz Pejhan, Mahnaz Jamee

**Affiliations:** 1 Pediatric Respiratory Diseases Research Center, National Research Institute of Tuberculosis and Lung Diseases (NRITLD), Shahid Beheshti University of Medical Sciences, Tehran, Iran; 2 School of Medicine, Islamic Azad University of Medical Sciences, Tehran, Iran; 3 Immunology and Allergy Department, Mofid Children’s Hospital, Shahid Beheshti University of Medical Sciences, Tehran, Iran; 4 Laboratory of Human Genetics of Infectious Diseases, Necker Branch, INSERM U1163, Paris, France; 5 Research Center for Immunodeficiencies, Pediatrics Center of Excellence, Children’s Medical Center, Tehran University of Medical Sciences, Tehran, Iran; 6 Lung Transplantation Research Center (LTRC), National Research Institute of Tuberculosis and Lung Diseases (NRITLD), Shahid Beheshti University of Medical Sciences, Tehran, Iran; 7 Tracheal Diseases Research Center (TDRC), National Research Institute of Tuberculosis and Lung Diseases (NRITLD), Shahid Beheshti University of Medical Sciences, Tehran, Iran; 8 Pediatric Infections Research Center, Research Institute for Children’s Health, Shahid Beheshti University of Medical Sciences, Tehran, Iran; 9 Laboratory for Pediatric Immunology, Department of Pediatrics, Willem-Alexander Children’s Hospital, Leiden University Medical Center, Leiden, Netherlands

**Keywords:** Hyper-IgE syndrome, Dominant-negative STAT3 mutation, Job’s syndrome, Lung surgery, Complications, Bronchopleural fistula

## Abstract

**Background:**

STAT3 hyperimmunoglobulin E syndrome (STAT3-HIES) also referred to as autosomal dominant HIES (AD-HIES) is an inborn error of immunity characterized by the classic triad of eczema, frequent opportunistic infections, and elevated serum IgE levels. As a consequence of lung sequels due to repeated infections and impaired tissue healing, patients may require interventional pulmonary procedures.

**Method:**

Four patients with dominant-negative *STAT3* mutations who had received interventional pulmonary procedures were enrolled. The demographic, clinical, and molecular characteristics were gathered through a medical record search. All reported STAT3-HIES patients in the literature requiring pulmonary procedures as part of their treatment were reviewed.

**Result:**

Recurrent episodes of pneumonia and lung abscess were the most prevalent symptoms. The most common non-immunological features were scoliosis, failure to thrive, and dental problems such as primary teeth retention and disseminated decays. Bronchiectasis, lung abscess, pneumatocele, and cavitary lesion were the most prevalent finding on high-resolution computed tomography at the earliest recording. All patients underwent pulmonary surgery and two of them experienced complications.

**Conclusion:**

Patients with STAT3-HIES have marked pulmonary infection susceptibility which may necessitate thoracic surgeries. Since surgical procedures involve a high risk of complication, surgical options are recommended to be utilized only in cases of drug resistance or emergencies.

## Introduction

Hyper immunoglobulin E (IgE) syndrome (HIES) is an inborn error of immunity with an annual incidence of 1 in 100,000 people, which was initially described in 1966 [1, 2]. A high level of serum IgE is immunologic characteristic of several autosomal recessive IEIs, namely interleukin 6 (IL-6) receptor deficiency, IL-6 signal transducer deficiency, ZNF431 deficiency, DOCK8 deficiency, PGM3 deficiency, CARD11 deficiency, CARD14 deficiency, and Comel-Netherton syndrome [3, 4]. However, Job’s syndrome, the prototype of HIES (AD-HIES), arises from a dominant negative mutation of the *STAT3* gene (signal transducer and activator of transcription 3) [5–7]. STAT3 is a member of the JAK-STAT pathway, which is involved in the differentiation and function of TH17. Abnormal Th17 function results in susceptibility to fungal and bacterial infections, particularly in the skin and respiratory system [8, 9]. Most patients present with the triad of eczema, frequent opportunistic infections, and elevated serum IgE levels. Moreover, non-immunological findings include facial characteristics, skeletal and dental abnormalities, vasculopathy, and malignancy [7–9]. Cutaneous involvement may present as skin abscesses and eczematous rashes. Abscesses commonly affect the face, neck, and scalp in the early years of life; they may not develop the typical signs and symptoms of infection and manifest as “cold “abscesses. The most prevalent pathogens are *Staphylococcus aureus* and *Candida albicans*. Papulopustular and eczematoid rashes first appear on the face and scalp in early infancy and progress to the upper trunk [10–13]. Sinopulmonary infections mostly caused by *Staphylococcus aureus*, *Streptococcus*, and *Haemophilus* species are prevalent and potentially fatal. Bronchitis and pneumatoceles are common complications of abnormal healing after lung infection which can be complicated due to secondary infection with *Pseudomonas aeruginosa* and *Aspergillus fumigatus* [1–5, 8, 14–16]. High levels of serum IgE (> 2,000 IU/uL) and eosinophilia are frequently observed in patients, however, increased serum IgE and eosinophilia do not predict the severity of the disease [1]. The IgE level tends to decrease or even reaches a normal level as age progresses. Low proportions of memory B and T cells associated with reduced IL-17A-producing T cells are also found in patients [1, 10].

General management goals in patients with STAT3-HIES include prophylaxis with antimicrobials, aggressive treatment of infection, and management of eczematoid dermatitis [17]. As a consequence of lung sequels due to repeated infections and impaired tissue healing, patients may require interventional pulmonary procedures such as abscesses drainage, lobectomy, or segmental lung resection because of the persistent pneumatoceles. Herein, we investigate the application of interventional pulmonary procedures and their outcomes in patients with hyper IgE syndrome.

## Method

The study was performed at the National Research Institute of Tuberculosis and Lung Diseases (NRITLD), Masih Daneshvari Hospital, Tehran, Iran, which is the national referral center for patients with inborn errors of immunity and pulmonary complications. The medical records of all patients with a clinical diagnosis of hyper IgE syndrome based on the European Society for Immunodeficiencies (ESID) diagnostic criteria and molecular diagnosis of dominant-negative *STAT3* mutations were investigated for a history of interventional pulmonary procedures. Follow-up of alive patients was performed via telephone interviews with patients.

The immunologic profiles of patients were determined by the measurement of complete blood count (CBC), lymphocyte subsets, serum immunoglobulin levels (IgG, IgA, IgM, and IgE), human immunodeficiency virus (HIV) serology, and lymphocyte transformation test (LTT).

Based on the clinical and laboratory findings, an NIH score was determined for each patient according to the National Institutes of Health (NIH) HIES rating system [18].

The DNA samples were sent to the Laboratory of Human Genetics of Infectious Diseases, France, and Research Center for Immunodeficiencies, Iran, and genetic studies by whole-exome sequencing (WES) were performed to identify underlying genetic defects.

A questionnaire was prepared and filled with patients’ data including demographics, molecular and clinical features. All statistical analyses were performed using SPSS software (v. 26.0, Chicago, IL).

Written informed consent for participation in this study was obtained from patients or their parents. This study was performed in accordance with the Declaration of Helsinki and was approved by the Ethics Committee of Shahid Beheshti University of Medical Sciences (Approval Code: IR.SBMU.NRITLD.REC.1400.049).

The literature search for reported STAT3-HIES patients with interventional pulmonary procedures was conducted in PubMed, Web of Science, and Scopus, applying the following keywords: “*STAT3* Loss-of-Function Mutation”, “*STAT3* Germ-Line Mutation signal transducer and activator of transcription 3 Loss-of-Function”, “dominant-negative *STAT3*”, “Mutations in *STAT3*”, “STAT3 deficiency”, “*STAT3*-LOF”, “Hyper-IgE syndrome”, “AD-HIES”, “Job's syndrome”, in combination with subsequent terminology: “surgery”, “intervention”, “drainage”, “thoracotomy”, “lobectomy”, “resection”, and “surgical treatment”. Reference lists of all full-text articles and major reviews were manually searched for additional studies.

## Result

### Summary of findings in study population

Four Iranian patients (3 males and 1 female) from four different families were enrolled, all of whom had a clinical and molecular diagnosis of dominant-negative *STAT3* mutation and a history of interventional pulmonary surgery (Table 1). The NIH scores of all patients at the time of diagnosis exceeded 40. All patients except P4 had consanguineous parents and none of them had a family history of STAT3-HIES. The disease first manifested at a median (IQR) age of 0.1 (0.1–0.5) years, while the clinical diagnosis of HIES was confirmed at a median (IQR) age of 12.0 (8.0–17.0) years after a median (IQR) delay of 11.9 (16.5–7.9) years.

**Table 1:** Summary of studies reported STAT3-HIES patients with lung surgical interventions

*NO*	*Article, Publication year*	*Country*	*# Of patients received lung surgery*	*Sex*	*Clinical and Molecular diagnosis*	*Age at the time of surgery (years)*	*Lung disorders (Indications for lung surgery)*	*Types of surgery*	*Complications of lung surgery*	*Outcome*
1	This article, 2023	Iran	4	M	*STAT3 (c.1909G* > *A, p.V637M)*	3, 5, 7	Pulmonary abscess (3 y/o and 5 y/o), Pulmonary empyema (Acinetobacter), bronchiectasis, aspergillosis infection	Lower right lung lobectomy (3 y/o), surgical drainage (5 y/o), surgical drainage and window insertion (Eloesser flapped surgery) (7 y/o)	Bronchopleural fistula	Alive
				M	*STAT3 (c.1909G* > *A, p.V637M)*	25.9	Hemoptysis (24 y/o), bronchiectasis (24 y/o), lung cavitary lesions (24 y/o), aspergillosis infection, Pseudomonas and Acinetobacter infection, abscess	Tube thoracostomy, thoracotomy	None	Deceased (25.9 y/o, Pulmonary abscess)
				M	*STAT3*	18.7	Pneumonia, croup, empyema, abscess, pneumatocele, bronchiectasis	Thoracotomy & decortication	Surgical wound infection	Alive
				F	*STAT3 (c.1397A* > *C; p.N466T)*	12	Lung abscess, extensive cystic bronchiectasis, pneumatocele, Pseudomonas infection	Thoracotomy	None	Deceased (15.7, Massive hemoptysis (despite angioembolization)
2	Harrison et al., 2021 [19]	UK	3 (P1, P4, P5)	M	*STAT3* (c. 1909G > A, p.V637M)	< 7y	Significant pulmonary infection (complicated cystic bronchiectasis with Pseudomonas infection and widespread Aspergillus infection leading to pulmonary HTN)	Left upper and lower lobectomies, Surgical drainage of infectious pericarditis	Bronchopleural fistula, Prolonged air leak, Exacerbation of scoliosis	Alive, Cessation of RTI following HSCT, Severe impairment of respiratory capacity after spinal surgery, Reliant on overnight CPAP and can ambulate only short distances
				M	*STAT3* (c. 1144C > T, p.R382W)	< 14y	Bronchiectasis, Severe multicystic and suppurative lung disease	Left upper lobectomy	-	Alive, Widespread decrease in number and size of cysts following HSCT
				F	*STAT3* (c. 1144C > T, p.R382W)	13y	Multiple abscesses and pneumatoceles	Right upper lobectomy	Bronchopleural fistula	Alive, Impaired pulmonary function before HSCT, aside from Aspergillus pericarditis post-HSCT, dramatic improvement in lung appearance after HSCT
3	Toribio-Dionicio et al., 2021 [20]	Peru	1	F	*STAT3* (c. 1144C > T, p.R382W)	2y, 4y and 8 y	Recurrent pneumonias, Empyema, Right pneumatocele, Acute respiratory failure, Left pneumothorax, Hemoptysis	Tube thoracostomy and thoracocentesis, Right pneumatocele resection, Left pleuropulmonary decortication and bullectomy, Surgical closure of the cutaneous bronchopleural fistulas	Bronchopleural fistulas, Right pneumatocele resection,	Alive, Decreased frequency of infections and hospitalizations
4	Frede et al., 2021 [21]	Germany	4	NA	*STAT3(*c.2125A > G, p.K709E)	NA	Recurrent pneumonia, abscess, pneumatocele	Right pneumonectomy	None	NA
				NA	*STAT3 (*c.1144C > G, p.R382G)	NA	Recurrent pneumonia, bronchiectasis, pneumatocele	Right upper lobe resection	None	NA
				NA	*STAT3 (*c.1723 T > G,,p.Y575D)	NA	abscess	Pneumonectomy	None	NA
				NA	*STAT1* (c.1154C > T p.T385M)	NA	Recurrent pneumonia and bronchiectasis	Left lower lobe resection	None	Passed away due to hepatopulmonary syndrome
5	Adatia et al., 2020 [22]	Canada	1	F	STAT3(c.1418C > T; p.A473V)	20	Pulmonary Abscess, Many episodes of pneumonia	Pneumonectomy	None	Alive
6	Fang et al., 2021 [23]	China	1	M	STAT3 (c.2137G > A, p.V713M)	6,9	Lung abscesses	Two lung surgeries for the treatment of lung abscess	None	Alive
7	Gonzalez-Mancera et al., 2020 [24]	USA	1	M	STAT3 (c.2144C > T; p.P715L)	28,33	Recurrent bronchitis, Pneumonia complicated by right sided empyema, Fibrothorax	Surgical drainage and pleurodesis (28 y/o), bilateral thoracotomy, Pleurectomy and decortication (33 y/o)	Hospital associated pneumonia, apical pulmonary fibrosis	Alive
8	Kandilarova et al., 2020 [25]	Bulgaria	1	F	*STAT3 (*c. 1144C > T, p.R382W*)*	1.5	Pneumonia with pleural empyema	Lobectomy	None	Alive (Worsening of the pneumatocele complicated with abscess in seventh year follow-up)
9	Zhang et al., 2020 [26]	China	1	F	*STAT3* (C.92G > A, p.R31Q)	34	Recurrent cough, dyspnea, shortness of breath, multiple bullae in both lung, pleurisy	Several surgical treatments for suspected pleurisy	None	Alive
10	Danion et al., 2020 [27]	France	2	M	*STAT3* (c. 1144C > T, p.R382W)	< 12y	Recurrent bacterial bronchopulmonary infections, Uncontrolled lung abscess, Bronchiectasis	Lower left lobectomy	-	Alive, ABPA episodes and unable to clear A. Fumigatus
				M	*STAT3* (c.1282-89C > T)	20y and 26y	Excavated opacities in the right upper pulmonary lobe, Azole-resistant A. Fumigatus infection	Lung wedge resection, Left upper lobectomy	Pneumothorax after first surgery	Alive
11	Kröner et al., 2019 [15]	Germany	9 (P5, P6, P7, P8, P9, P10, P11, P12, P13)	F	*STAT3* (p.R382Q)	4y and 5y	Pneumonia, Hemoptysis, Bronchiectasis, Architectural distortion, Caverna, Emphysema	Pneumectomy, Lobectomy	Post-operative prolonged pneumonia by C. albicans and S. aureus (*n* = 1), acute A. fumigatus infection (*n* = 2), persistent empyema (*n* = 3), Bronchopleural fistula (*n* = 4)	Deceased (16.8 y/o)
				M	*STAT3* (p.R382W)	15y	Repeated bronchitis, Hemoptysis, Bronchiectasis, Architectural distortion, Pneumatocele, Cysts	Abscess drainage		Alive
				M	*STAT3* (p.R382Q)	3y	Pneumonia, Chronic cough, Dyspnea, Hemoptysis, Bronchiectasis, Architectural distortion, Pneumatocele	Lobectomy		Alive
				F	*STAT3* (p. Q469R)	4y and 9y	Pneumonia, Chronic cough, Hemoptysis	Lobectomy, Bilobectomy		Alive
				F	*STAT3* (p.R382Q)	13y, 20y, and 21y	Repeated bronchitis, Chronic cough, Hemoptysis, Bronchiectasis, Architectural distortion, Multiple cysts, Pleural thickening	Decortication, Pleurectomy, Pneumectomy		Alive
				F	*STAT3* (p.V713L)	4y	Pneumonia, Dyspnea, Hemoptysis, Bronchiectasis, Architectural distortion	Lobectomy		Alive
				M	*STAT3* (p.R382W)	24y	Hemoptysis, Bronchiectasis, Architectural distortion, Large caverna, Cysts, Centrilobular emphysema	Bilobectomy		Deceased (28.8 y/o)
				M	*STAT3* (p.F348L)	1.5y, 21y, 24y	Chronic cough, Pneumonia, Dyspnea, Small bullae, Cicatrization, Pleural thickening	Lobectomy, Abscess drainage, Pleurolysis, Lobectomy		Alive
				F	*STAT3* (p.R382W)	9y	Chronic cough, Repeated bronchitis, Dyspnea, Hemoptysis, Bronchiectasis, Architectural distortion, Cysts	Abscess drainage		Alive
12	Duréault et al., 2019 [28]	France	6	-	-	-	Aspergilloma (*n* = 2), pneumothorax (*n* = 1)	lobectomy (*n* = 1), wedge resection with pleurectomy (*n* = 1)	CCPA 30 months after surgery with complications localized to the same territory as the aspergilloma	All Alive
							Chronic cavitary pulmonary aspergillosis (*n* = 4)	lobectomy (*n* = 3), segmentectomy (*n* = 1), pleurectomy (*n* = 1), Drainage of purulent pneumothorax (*n* = 1), pleuroscopy and pleurodesis (*n* = 1)	Pneumothorax (*n* = 2), relapse of lung aspergillosis (*n* = 3)	
13	Fan et al., 2018 [29]	China	2	F	STAT3 (c.1144C > T, p.R382W)	4, < 7	Lund abscesses, Cyst forming pneumonia, cavitation	lobectomy	None	Alive
				F	STAT3	3	Granulomatous formation of bronchi, Cystic structures in right upper lobe, H. influenza and Aspergillus infection	Close thoracic drainage	None	Alive
14	Ahmed et al., 2016 [30]	USA	1	F	STAT3(c.1962_1964delCAT)	18	Large pneumatocele	Partial pneumonectomy	None	Alive
15	Gernez et al., 2016 [31]	USA	1	M	STAT3 (1144C > T, p.R382W)	3,22	Recurrent pneumonia, pneumatocele Hemoptysis, mycetoma, bronchiectasis, aspergillus infection,	Right upper lobectomy, Right pneumonectomy	None	Alive
16	Zegarra-Linares et al., 2014 [32]	USA	1	M	STAT3	15	Hydropneumothorax, left lower lobe cavitation,	video-assisted thoracic surgery with chest tube insertion and left Lowe lobectomy	Persistence of hydropneumothorax after video –assisted thoracic surgery	Alive
17	Freeman et al., 2013 [33]	USA	32	F	STAT3 (p.V637M)	8	Recurrent pneumonia (S.aureus)	Wedge resections,	Persistent bronchopleural fistula	Alive; required revision surgery
						NA	Post surgical bronchopleural fistula	Revision surgeries; right upper lobe resection; chest wall revision with thoracoplasty and muscle flap coverage	–––	
				F	STAT3 (p.F384S)	9	Pneumatocele	Wedge resection,	Bronchopleural fistula with S. aureus empyema	Alive; required revision surgery
						NA	Post surgical bronchopleural fistula	Revision surgery; muscle flap repair	–––––	
				F	STAT3	26	Aspergillus infection	Wedge resection,	Persistent bronchopleural fistula complicated by Eikenella corrodens, S. aureus, and Aspergillus, empyema with S aureus and Aspergillus	Alive; required revision surgery
						NA	Post surgical bronchopleural fistula and empyema	Revision surgeries; right pneumonectomy, Clagett window		
				M	STAT3 (p.R382Q)	30	Aspergillus infection	Lobectomy,	Bronchopleural fistula	Alive
				F	STAT3 (p.G618D)	9	Extensive bronchiectasis	Bilobectomy	Bronchopleural fistula and prolonged healing of the chest tube site	Alive
						21	Aspergillus spp	Wedge resection	–––––	Alive
				F	STAT3	29	NA	Lobectomy	Bronchopleural fistula and prolonged healing of the chest tube site	Alive
				F	STAT3 (p.R382W)	23	P. aeruginosa infection	Lobectomy	Bronchopleural fistula with secondary empyema	Passed away after 10 months due to respiratory failure
						NA	Post surgical bronchopleural fistula and empyema	Revision surgery; right middle lobe resection		
				F	STAT3 (p.Q644del)	5	Persistent pneumonia	lobectomy	Bronchopleural fistula	Alive
				F	STAT3 (p.N472)	31	Pneumatocele	Wedge resection	Bronchopleural fistula and Aspergillus empyema	Alive
				M	STAT3 (p.R382W)	17	Pneumatocele	Lobectomy	Bronchopleural fistula	Alive
				M	STAT3 (p.F384L)	22	Pneumatocele (S.aureus)	Wedge resection	bronchopleural fistula and empyema	Alive, He underwent wedge resection twice due to recurrent pneumatoceles and after both times required revision surgeries
						NA	Post surgical *bronchopleural fistula* and empyema	Revision surgery; Pleurolysis and drainage	––––	
						24	Pneumatocele (S.pneumoniae)	Wedge resection	*Bronchopleural fistula* and Aspergillus empyema	
						NA	Post surgical *bronchopleural fistula* and Aspergillus empyema	Revision surgery; empyema, debridement, right upper lobe resection	–––-	
				M	STAT3 (p.R382W)	23	Multiple abscesses	Bi-lobectomy	Progressive pulmonary failure with aspergillus, pseudomonas and staphylococcus	Passed away due to progressive pulmonary infection 5 years later
				M	STAT3 (p.R382Q)	9	Aspergillus virinidutans infection	Partial lobectomy	Aspergillus viridinutans empyema	Passed away due to chronic complications of chronic pulmonary Aspergillus infection
						Na	Post surgical Aspergillus viridinutans empyema	Revision surgeries; Multiple chest tube drainage, thoracotomies, debridement, intrapleural antifungals and granulocyte		
				M	STAT3 (p.P639T)	2	Recurrent pneumonia	Bi-lobectomy	––-	Alive
						9	pneumatocele	Wedge resection	*Bronchopleural fistula*	Alive
				M	STAT3 (p.V637M)	15	Pneumatocele (P. aeruginosa & Aspergillus unguis)	Wedge resection	*Bronchopleural fistula* from numerous parenchymal sites	Alive required revision surgery
						NA	Post surgical *bronchopleural fistula*	Revisions surgery; prolonged chest drainage, oversewing of air leakage, biologic glue and pleural tent	–––-	
				M	STAT3 (p.S611G)	4	Aspergillus pneumonia	Lobectomy	*Bronchopleural fistula* with empyema	Alive, required revision surgery
						NA	Post surgical *bronchopleural fistula* and empyema	Biologic glue		
				F	STAT3 (p.V637M)	2	Pneumatocele (S.aureus)	Wedge resection	*bronchopleural fistula* with S.aureus empyema	Alive
				F	STAT3 (p.R382Q)	20	Multiple abscesses	Bi-lobectomy	*bronchopleural fistula* with Aspergillus empyema	Alive, required revision surgeries
						NA	Post surgical *bronchopleural fistula* with Aspergillus empyema	Revision surgery; right middle lobe/right upper lobe resection and right pneumonectomy	––––––	
				F	STAT3 (p.E652K)	5	NA	NA	NA	NA
				M	STAT3 (p.T622I)	10	S.aureus infection	Left lung resection	None	Alive
				F	STAT3 (p.V637M)	3	Mold infection	Right middle lobectomy	None	Alive
				M	STAT3 (p.V637M)	7	Recurrent infections	lobectomy	None	Alive
				M	STAT3 (p.F384L)	1.5	Pneumatocele (S.aureus)	Lobectomy	None	Alive
				M	STAT3 (p.T341N)	47	Aspergillus spp infection	Thorascopic wedge resection	None	Alive
				M	STAT3 (p.Y657C)	33	Histoplasmosis infection	Lobectomy	None	Alive
				F	STAT3 (p.Q644del)	2	Pneumatocele	Wedge resection	None	Alive
				F	STAT3 (p.R382W)	23	S.aureus infection	Lobectomy	None	Alive
				F	STAT3 (p.Q644del)	2	Pneumatocele	Wedge resection	None	Alive
				F	STAT3 (p.V637M)	15	Necrotizing pneumonia	Wedge resection	None	Alive
				M	STAT3 (p.R382Q)	3	Pneumatocele	Wedge resection	None	Alive
				M	STAT3 (delAA371-380)	26	Persistent hemoptysis, extensive bronchiectasis	Lobectomy	None	Alive
				M	STAT3 (p.W479C)	45	Aspergillus spp infection	Wedge resection	None	Alive
				F	STAT3 (p.W479C)	44	Aspergillus spp and pseudomonas aeroginosa	Lobectomy	None	Alive
18	Zhang et al., 2013 [34]	China	2	NA	STAT3	NA	Lung abscess, pneumatocele (*n* = 2)	Lobectomy (*n* = 2)	None	Alive
19	Sterbank et al., 2013 [35]		1	M	STAT3 (p.T389I)	2	Empyema	lobectomy	None	Alive
20	Robinson et al., 2011 [36]	USA	1	M	STAT3 (c.1772A > T, p.K591M)	< 3	Respiratory distress, pneumonia, pneumatocele, parapneumonic effusion	Left thoracotomy with bleb plication/pleurodesis	None	Alive
21	Liu et al., 2010 [37]	China	1	M	STAT3 (c.1970A > C, p.Y657S)	3	Staphylococcal pneumonia, multiple pulmonary abscesses, right pyo-pneumothorax, pneumatocele	Surgical drainage	None	Alive
22	Xie et al., 2010 [38]	China	1	M	*STAT3* (c. 1310A > C, p. H437P)	8y	Recurrent pneumonias, Ten years history of productive cough and one year history of bloody sputum	Right upper lung cyst surgical therapy	At 20 y/o, presented with bilateral cystic change, extensive consolidation in right and patchy infiltration in left with S. aureus identified in BAL	Alive

*ABPA* Allergic bronchopulmonary aspergillosis, *CPAP* Continuous positive airway pressure, *F* Female, *HSCT* Hematopoietic stem cell transplantation, *HTN* Hypertension, *IQR* Interquartile range, *M* Male, *NA* Not available, *RTI* Respiratory tract infection, *Spp.* Several species, *y/o* year (s) old

Recurrent episodes of pneumonia and lung abscess were the most prevalent symptoms (4/4). Three out of four patients (P1, P2, P4) developed pulmonary mold infection (Aspergillus was the most common organism). Moreover, non-pulmonary infections such as chronic diarrhea (P2, P3), otitis media, rhinosinusitis, conjunctivitis (P1), mucocutaneous candidiasis (P1, P3) and lymphadenitis (P1) were also detected. Skin infection, eczema and newborn rash were the most common skin manifestations presented in all patients. Food allergy was detected in 2 patients (P1, P3).

The most common non-immunological features were scoliosis (P2, P3, P4), failure to thrive (FTT) (P3, P4) and dental problems such as primary teeth retention (P2, P3) and disseminated decays (P2). Mandibular surgery due to a dental problem was performed in P2. Other non-immunological features contained clubbing, enteropathy, anemia (P2), oral ulcers (P3) and hyperextensible joints (P1).

Malignancy, chronic *Epstein–Barr virus* (EBV) infection and major bleeding or thrombotic vascular event were not found in any of the patients during the median (IQR) 4.75 (6.15–3.8) years of follow-up.

Immunological workup was performed in all 4 patients. The most prominent finding was the elevated serum IgE level and eosinophil count (4/4). White blood cell (WBC) count and lymphocyte subpopulations were normal in all patients except P3 who had increased CD19 + B cell count. High levels of IgG and IgM were detected in 3 patients (P1, P3, P4), while all had normal levels of IgA. The most prevalent findings on high-resolution computed tomography (HRCT) at the earliest recording were bronchiectasis (4/4), lung abscess (4/4), pneumatocele, and cavitary lesion (2/4). All patients underwent pulmonary surgery and P1 (bronchopleural fistula) and P3 (wound infection) experienced complications. Four patients received antibacterial and antifungal prophylaxis along with intravenous immunoglobulin (IVIG) (except P3). Two patients (P2 and P4) died due to pulmonary arrest and massive hemoptysis.

### Detailed description of patients

#### Case 1

The patient was a 10-year-old male born to consanguineous parents with a history of eczematous dermatitis from the age of 10 days, which had extended to the face and diaper area. He had a productive cough and fever at the age of 3 years, which led to the diagnosis of pulmonary abscess and he underwent drainage and lower right lung lobectomy. He developed a cough and fever when he was 4 years old. Physical examination revealed hyperextensible joints. Laboratory findings at the age of 4 showed normal WBC count, increased eosinophil count (15%), IgE (532 (IU/ml)) and IgG level (2160 (IU/ml)). Lymphocyte subpopulations (CD3 + , CD4 + , CD8 + T cells, CD19 + B cell, natural killer (NK) cell) were normal. Bronchoalveolar lavage** (**BAL) culture was positive for *Aspergillus terreus* and *Acinetobacter*. Moreover, chest X-ray (CXR) demonstrated lung cavity formation in right lower lobe, pneumothorax, pleural effusion on the right side, and necrotizing pneumonia with empyema. According to the National Institutes of Health (NIH) HIES rating system [18], he scored 49 and the diagnosis of HIES was made. At the age of 5 years, he was presented with a fever and cough. The Chest sonography was suggesting for infectious lesion (abscess or empyema) and he underwent drainage and medical treatment. He was referred to the hospital again with cough and fever at the age of 7 years due to pulmonary empyema with *Acinetobacter* species. A thoracostomy window was opened for the patient from the back of the chest (Eloesser flapped surgery). Bronchopleural fistula occurred as a complication of surgery. He has received monthly IVIG and antimicrobial prophylaxis with tavanex, cephalexin and voriconazole. He responded well and the frequency of infections has reduced.

#### Case 2

The patient was a 26-year-old boy born to consanguineous parents. His family history was unremarkable. His medical history was notable for newborn rush, recurrent skin abscess, eczematous dermatitis, FTT, bronchiectasis and scoliosis. The HIES was suspected based on laboratory findings and clinical features. Laboratory findings at the age of 22 years revealed increased eosinophil count (610 × 10^3^ (cells /µL)) and IgE level (> 1250 (IU/ml)). However, WBC count, lymphocyte subpopulations (CD3 + , CD4 + , CD8 + T cell, CD19 + B cell and NK cell) and serum level of immunoglobulins (IgG, IgA, IgM) were normal. Non-immunological findings included clubbing, chronic diarrhea, and anemia. According to the NIH HIES rating system, he scored 46. The diagnosis of HIES was confirmed by the identification of *STAT3* missense mutation when he was 23 years old. Dental problems such as primary tooth retention, disseminated decay and abscess led to mandibular surgery. He developed pneumonia, hemoptysis, bronchiectasis, lung cavitary lesions and pneumatocele at the age of 24 years. In addition, *Pseudomonas*, *Acinetobacter* and pulmonary mold infection with *Aspergillus* were reported on BAL culture. CXR showed pneumatocele and HRCT revealed bronchiectasis, abscess and lung cavitary lesion. He underwent tube thoracostomy and thoracotomy due to permanent infection and empyema when he was 26 years. Although he received antimicrobial prophylaxis with vancomycin, tazocin, amikacin, tobramycin, colistin, meropenem, voriconazole and itraconazole, unfortunately, he died due to pulmonary arrest at the age of 26 years.

#### Case 3

The patient was a 23-year-old boy born to consanguineous parents. His family history was unremarkable. He had a history of newborn rash, eczematous dermatitis since 1-month-old, primary teeth retention, scoliosis, FTT, oral ulcer, food allergy and recurrent respiratory tract infections (pneumonia, croup, bronchiectasis). He was hospitalized due to croup at the age of 6 months and a skin abscess at the age of 13 years. Laboratory findings at the age of 12 years revealed normal WBC count, elevated eosinophil (1410 (cell/μL)) and CD 19 + B cell count (720 × 10^3^ (cells /µL)) accompanied by high IgG (1758 mg/dl), IgE (800 IU/ml) and IgM (234 mg/dl) level. However, the IgA level and other lymphocyte subpopulations (CD3 + , CD4 + , CD8 + T cells, NK cell) were normal. DEXA scan showed normal bone densitometry and according to the NIH HIES rating system, he scored 47. Based on clinical features and laboratory findings the diagnosis of HIES was made at the age of 12 years old. At 18 years old he was referred to the hospital for fever and cough. CXR demonstrated cavitary lesion and pleural effusion. Moreover, HRCT findings revealed cavitary lesions, pleural effusion, pneumatocele, lung abscess and bronchiectasis. He underwent thoracotomy and decortication surgery due to pulmonary abscess and empyema which was further complicated by wound infection but responded to antibiotic therapy.

He received antimicrobial prophylaxis with vancomycin, imipenem, cotrimoxazole, and itraconazole and responded well.

#### Case 4

The patient was a 15-year-old girl born to non-consanguineous parents. Her family history was unremarkable. She had a history of newborn rash, eczematous dermatitis, skin abscess since she was 45 days old, scoliosis and FTT. In addition*,* multiple hospitalizations due to recurrent lung infections and lung abscesses were reported. At the age of 12, she was hospitalized due to pneumonia. Laboratory findings revealed increased eosinophil count (800 × 10^3^ (cells /µL)), IgG (1805 mg/dl), IgM (199 mg/dl) and IgE level (4848 IU/ml). BAL culture was positive for *Pseudomonas aeruginosa*. Moreover, HRCT findings were suggestive of extensive cystic bronchiectasis, abscess and pneumatocele. CXR demonstrated multiple cavities. According to the NIH HIES rating system, she scored 55 and the diagnosis of HIES was confirmed based on clinical features, laboratory findings and identification of missense *STAT3* mutation. She underwent a thoracotomy due to a lung abscess when she was 12 years old. She received pulmonary physiotherapy, IVIG and antimicrobial prophylaxis with colistin, vancomycin, meropenem, cotrimoxazole, fluconazole and amphotericin B, but unfortunately, she died due to massive hemoptysis at the age of 15 years.

### Summary of the literature review

A total of 22 articles reported 77 cases of STAT3-HIES patients who received lung surgery for the management of pulmonary infections from 2010 to 2023 (Table 1). Gender was reported in 66 cases and included 35 male and 31 female. The median (IQR) age of patients at the time of first surgery was 9.5 (3.0–22.3) years. Lobectomy was the most frequent type of surgery performed in 45 patients (58.4%), followed by wedge resection (17, 22.1%), surgical drainage (12, 15.6%) and pneumonectomy (11, 14.3%). Six patients (7.8%) had thoracotomy. Other surgeries consisted of decortication (5, 6.5%), pleurectomy (4, 5.2%), thoracostomy (3, 3.9%), pleurodesis (3, 3.9%), window insertion (2, 2.6%), muscle flap repair and segmentectomy (1, 1.3%). For three patients the type of lung surgery was not specified (3.9%). Need for second surgery was reported in 21 patients (27.3%). Bronchopleural fistula was the most common complication after surgery (24, 31.2%). Seven patients experienced empyema (9.1%). Pneumonia and prolonged healing of wound were observed in 4 (5.2%) and 2 (2.6%) patients, respectively. Eight (10.8%) patients had deceased outcome.

## Discussion

In this study, we presented four patients with STAT3-HIES receiving various types of interventional pulmonary procedures and searched the literature for similar patients. Recurrent pulmonary infections are frequently observed in STAT3-HIES patients since childhood. Surgical procedures are utilized to manage lung infections and complications such as the formation of pneumatoceles which are prone to secondary infection, abscess and empyema. The number of large cohorts evaluating the impact of interventional lung procedures in STAT3-HIES patients is limited, three of which is discussed in more details.

Post-operative sequels are often reported due to underlying dominant-negative *STAT3* mutations and wound-healing abnormalities [15]. In the present study, bronchopleural fistula and wound infection occurred in two patients after surgery. In a retrospective study by Freeman et al. on 32 STAT3-HIES patients receiving 36 pulmonary surgeries, surgical complications were observed in more than half of all procedures. The most frequent complication, occurring in 47% of surgeries, was bronchopleural fistula which frequently led to empyema. Among complicated surgeries, 47% required further surgery due to long-standing bronchopleural fistula and empyema. It was suggested to prioritize medical treatment and only select surgical options in cases with ineffective medical treatment and significant symptoms [33].

In the same manner, in another study on nine patients with STAT3-HIES, Kroner et al. observed high rates of post-surgical complications, declined functional lung tissue, and negative impact on the long-term outcome. They recommended strict monitoring of pulmonary function and regular inhalation therapy with hypertonic saline, chest physiotherapy, airway clearance techniques, workout programs, and using intensive therapy for controlling infections (i.e., continuous use of oral antibiotics against S. aureus, regular antibiotic therapy in patients with high rates of pulmonary exacerbations, intense IV antibiotic therapy of exacerbations using fluoroquinolones) [15].

In the review of the literature, almost one-third of patients (27.3%) required more than one episode of lung surgery. The pre-surgery condition is an important determinant of surgical outcome. In accordance with previous studies, a history of infection with specific organisms was associated with a worse prognosis. Two out of four patients described in the current study suffered from *Aspergillus* infection and one of them required two other episodes of lung surgery. In a retrospective French study on STAT3-HIES patients with pulmonary Aspergillosis, six patients underwent seven episodes of surgery. The authors concluded that while many patients with HIES may develop aspergillosis infections, surgery can provide a permanent cure for pulmonary aspergillosis, but a recurrence might be inevitable [28].

None of the patients in the present study underwent hematopoietic stem cell transplantation (HSCT), although all patients received antimicrobial prophylaxis and IVIG and two of them responded well. Recently Harrison et al. in a cohort of patients with STAT3-HIES receiving HSCT suggested allogeneic HSCT as a curative measurement even in patients with prior severe lung disease. In their cohort, three patients had undergone lung surgeries before HSCT, but considerable improvement in lung disorders was only achieved after HSCT. None of them required lung surgery post-HSCT and no peri-transplant pulmonary inflammatory complications were observed [19].

This study faced several limitations including small number of participants and its retrospective nature. In addition, in the literature review some patients might have been included in more than one study, affecting the calculation of percentages. However, this study highlights the consequences of lung surgery in STAT3-HIES patients and the therapeutic dilemma for clinicians, which will be hopefully solved with future reports of more patients.

## Conclusion

Patients with STAT3- HIES are prone to frequent pulmonary infection and other infection-related complications which lead to lung tissue destruction and significant mortality or morbidity; therefore, prompt diagnosis and prophylaxis initiation are critical. Furthermore, thoracic surgeries in these patients carry a considerable risk of complications, thus surgical procedures are only recommended when medical treatment has failed or there are severe symptoms.

## Data Availability

The datasets used and/or analyzed during the current study are available from the corresponding author on reasonable request.

## Abbreviations

AD: Autosomal dominant
AR: Autosomal recessive
CBC: Complete blood count
DEXA: Dual-energy X-ray absorptiometry
EBV: Epstein–Barr virus
GOF: Gain of function
HIES: Hyper immunoglobulin E syndrome
HRCT: High-resolution computed tomography
HSCT: Hematopoietic stem cell transplantation
IGRT: Image-guided radiation therapy
NIH: National Institutes of Health
WBC: White blood cell
WES: Whole-exome sequencing
